# Anatomical landmarks and procedure technique of Levator Scapulae Plane Block (LeSP block): Case report: Ultrasound-guided block of the superficial, deep and distal planes of the levator scapulae muscle for treatment of shoulder girdle myofascial pain

**DOI:** 10.1016/j.radcr.2024.09.050

**Published:** 2024-09-26

**Authors:** Roberto Del Valhe Abi Rached, Leandro Ryuchi Iuamoto, Angela Hyun Ji Kim, Guilherme Yuiti Sikusawa, Fernanda Mayume Souza, Wu Tu Hsing

**Affiliations:** a Institute of Physical Medicine and Rehabilitation, Hospital das Clínicas, University of Sao Paulo School of Medicine, Sao Paulo, Brazil; b Center of Acupuncture, Department of Orthopaedics and Traumatology, University of Sao Paulo School of Medicine, Sao Paulo, Brazil

**Keywords:** Fascia, Neck pain, Chronic pain, Nerve block, Case report

## Abstract

Neck pain is a prevalent issue associated with musculoskeletal disorders. This study describes the interfascial Levator Scapulae Plane Block (LeSP Block) technique, using ultrasound guidance for local anesthetic administration to treat chronic neck pain. Two patients, 1 77-year-old female and 1 50-year-old female, underwent the LeSP Block. Immediate postprocedure pain relief was achieved in both, with 1 patient experiencing complete pain remission (VAS = 0) and the other showing significant improvement (VAS = 2) after 30 days. The LeSP Block demonstrated effectiveness and ease of use, suggesting its inclusion in pain management strategies for shoulder girdle and scapular pain. Further anatomical studies aimed at improving the anatomical description of the accessory spinal nerve are recommended to refine the technique.

## Introduction

Complaints of cervical, shoulder and interscapular pain are common in pain clinics and can significantly impact patients' work and quality of life [1,2]. Several etiologies are responsible for these complaints, such as spondylarthrosis, cervical discopathies, rotator cuff tendinopathies, and myofascial pain syndrome [[2], [3], [4], [5]].

Myofascial pain syndrome (MPS) usually does not respond to pharmacological or nonpharmacological treatments, reduces quality of life and can even cause problems such as anxiety and depression [2,6,7]. Several methods of controlling MPS have been developed in recent decades, including stretching exercises, strengthening, anesthetic injection of the trigger point, acupuncture, and intramuscular electrical stimulation [8,9].

It has recently been suggested that myofascial pain is best explained as secondary hyperalgesia of peripheral neural origin [10,11]. Based on this, the effect of interfascial block for treatment of pain in the shoulder girdle and in the interscapular region was evaluated at 2 different points, superficial and deep fascia of the levator scapula muscle, with the expectation of performing the block of the distal sensory branches of the accessory and dorsal spinal nerves of the scapula respectively [[12], [13], [14]].

The effectiveness of the fascial plane block mechanism may be due to a local effect on nociceptors and neurons within the plane or in adjacent muscle and tissue compartments, and the extension is due to dissemination. Analgesia and cutaneous sensory loss are not completely predictable due to anatomical variation, which governs fluid dispersion and the pharmacodynamics of local anesthetics. The other mechanism is local anesthetic vascular absorption and a systemic analgesic effect at distant sites [13].

Because of its proximity to the accessory spinal nerve path, the superficial fascia region was chosen. The XI nerve gives rise to the accessory spinal nerve. The accessory spinal nerve, along with the internal jugular vein, common carotid artery, and vagus nerve, exits the skull through the jugular foramen. It then crosses the carotid triangle of the neck before crossing through (63%) or below (37%) the sternocleidomastoid muscle [[15], [16], [17]]. The nerve emerges from the sternocleidomastoid muscle to enter the posterior triangle of the neck approximately 8 cm cranial to the clavicle [14] and then gives motor supply to the trapezius and sternocleidomastoid muscles. Despite its motor function being largely well documented, some studies have also demonstrated its role in nociception [18].

The deep fascia region was also chosen because of the anatomy of the dorsal nerve of the scapula, which is a motor nerve formed by the anterior branch of the C5 cervical root. It emerges from the scalenus medius muscle and passes between the levator scapulae and serratus posterior superior muscle along the medial border of the scapula, anterior to the rhomboids minor and major to finally provide innervation to these muscles. Despite being a motor nerve, it can cause pain in the interscapular region [19].

As a result, the hypothesis is that an interfascial block with local anesthetic guided by ultrasound (US) would alleviate interscapular and shoulder girdle pain. The current study's goal is to describe the anatomical and ultrasonographic parameters, as well as the technique for performing an interfascial block with local anesthetic guided by US (LeSP Block).

## Case presentation

The report of the rehabilitation of 2 consecutive cases treated at Instituto de Ortopedia e Traumatologia do Hospital das Clínicas da Faculdade de Medicina da Universidade de São Paulo (IOT-HCFMUSP) was carried out. Participants signed an informed consent form approved by the ethics committee of the School of Medicine of the University of São Paulo, according to the Declaration of Helsinki Ethical Principles for Medical Research Involving Human Subjects.

The study was approved by the local ethics committee.

The evolution of the cases was assessed before, immediately after and 30 days after the procedure.

The 2 study participants were already undergoing rehabilitation at the IOT-HCFMUSP because of myofascial pain in the region comprising the trapezius muscle, rhomboids and medial border of the scapula. According to anamnesis and physical examination performed by the same physiatrist, both had pain for more than 6 months, no diagnosis compatible with fibromyalgia, and no suspicion of rheumatologic disease, cervical radicular pain, or rotator cuff syndrome.

Case 1: The first patient, I.F.S., a 77-year-old woman, reported continuous pain despite having undergone 40 physical therapy sessions. Her medical history included heart failure caused by coronary artery disease, type 2 diabetes, systemic arterial hypertension, and previous treatments for breast and bowel cancer. The patient had right shoulder injuries, including marked subacromial-subdeltoid bursitis, pericapsular edema (inflammatory process), supraspinatus tendinopathy with partial bursal fiber tear (50% thickness), infraspinatus tendinopathy with longitudinal fissures (without full-thickness tears), diffuse glenoid labrum degeneration, and acromioclavicular osteoarthritis.

She described the pain as a burning sensation with stabbing and shock-like qualities, continuous in nature, and particularly intense upon waking, despite reporting that her mattress and pillow were adequate. Triggering factors included physical exertion, such as household chores and ironing, while worsening factors included sweeping and other domestic tasks. She reported partial relief with tramadol and noted slight edema in the shoulder, without hyperemia. To control her pain, the patient used tricyclic antidepressants, muscle relaxants, simple analgesics, and rescue opioids, along with specific medications for her comorbidities.

Her surgical history included a right breast quadrantectomy in 2007 for breast cancer, a 20 cm sigmoidectomy in 2015 for bowel cancer, bilateral cataract surgery in August 2020, external tibial fracture correction on her left leg following a car accident 35 years ago, and total knee arthroplasty on the left side in 2007.

On the day of the procedure, she had a pain score of VAS = 7, located in the shoulder girdle and right shoulder. On physical examination, tense bands and active trigger points were observed in the trapezius, levator scapulae, and right rhomboid muscles. After the fascial plane block of the levator scapulae muscle, the patient reported complete pain relief, with VAS = 0, both on palpation and active movement of the right shoulder, and did not present any neurological deficits, such as loss of strength in the right arm or hand, or difficulty swallowing. After 30 days, she was reevaluated by telephone and reported a mild recurrence of pain, with VAS = 2. The patient indicated that the pain had returned to baseline, possibly due to perpetuating factors of myofascial pain related to inadequate posture during domestic activities.

Case 2: The second patient, C.F.C., a 50-year-old radiology technician with hypercholesterolemia, presented with severe pain in the right shoulder girdle, exacerbated by professional activities such as raising her arm above shoulder height to position radiology films. She did not use pain medication. Her imaging revealed an acromioclavicular joint with regular contours and no subacromial spurs, as well as a slight thickening of the acromial insertion of the coracoacromial ligament. The humeral head had normal morphology and signal, and the other bony structures showed preserved morphology and marrow signal.

Additionally, the patient had subscapular tendinopathy with a small, low-grade partial intrasubstance tear, measuring approximately 0.4 cm, along with mild supraspinatus tendinopathy without tears. The infraspinatus and teres minor tendons had normal thickness, continuity, and signal. The long head of the biceps brachii tendon was centered in the bicipital groove and was intact in both intra- and extra-articular pathways. The glenoid labrum structures had regular contours and preserved signal without any lesions. Subacromial-subdeltoid bursitis was present, characterized by edema and bursal thickening. There was no glenohumeral joint effusion, and the muscle bellies showed normal trophism.

On physical examination, tense bands were identified in the trapezius, levator scapulae, and rhomboids, which reproduced her reported pain. She had no neurological deficits and reported a VAS score of 6 before the procedure. Following the fascial plane block, she reported complete pain relief (VAS = 0). After 30 days, during a follow-up phone call, she remained pain-free (VAS = 0).

### Description of procedure—LeSP block

The procedures were carried out in a procedure room at the XXXXXX. The technique was carried out without an adapter on the transducer for the free direction of the needle and adequate approximation to the anatomical target using a Venue Go® ultrasound device from the manufacturer GE healthcare® with a high-frequency 12L-RS linear transducer. Patients were seated with the upper limbs supported and the neck slightly flexed.

**Fig. 1:**
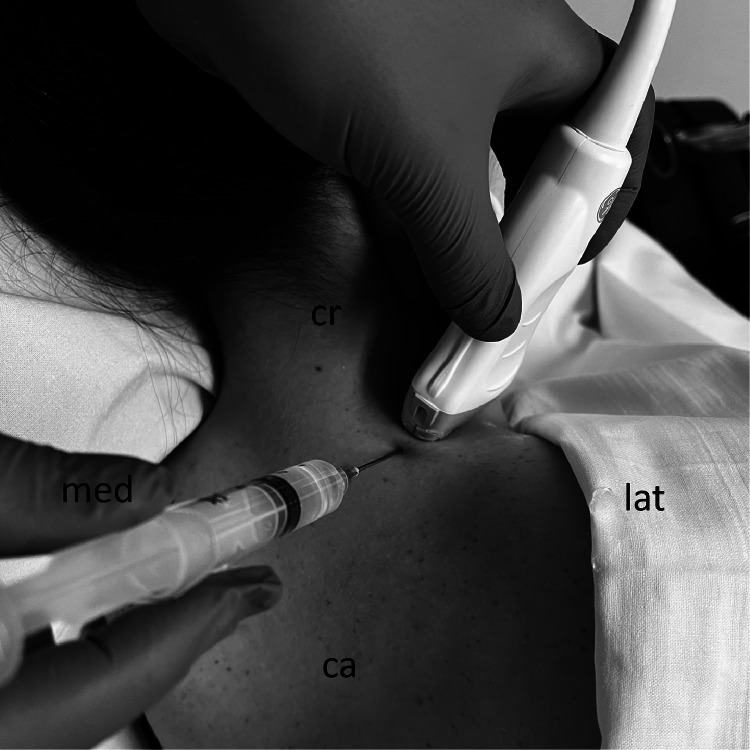
In-plane injection technique - Levator scapular plane (LeSP) block. ca, caudal, cr, cranial, med, medial, lat, lateral.

The syringe needle is oriented in plane to the transducer and introduced, with its tip between the muscular planes of the trapezius and levator scapulae and the second point between the levator scapula muscle visualized in the long axis and the second rib (Fig. 1). The injected substance must dissect this space to separate the muscles. For each injection point, 5ml of solution with 5% glucose (D5W) and 0.25% levobupivacaine was used. Likewise, 2 points can be sequentially performed, resulting in a total of 10 mL. To obtain the solution, 50% dextrose, water for injection and levobupivacaine was used and as a final result, 5% dextrose and 0.25% levobupivacaine was obtained.

**Fig. 2:**
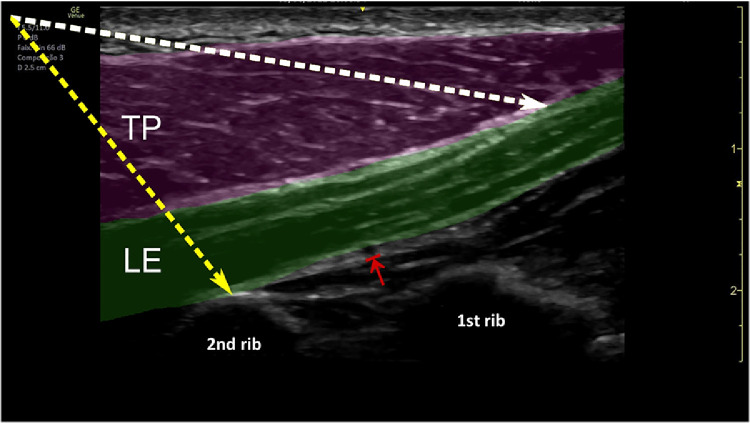
Levator scapular plane (LeSP) block in ultrasound view. TP, trapezius muscle in pink; LE, levator scapulae muscle in green; red arrow, dorsal artery of the scapula; white dashed line - LeSP 1 infiltration site; yellow dashed line - LeSP 2 infiltration site.

**Fig. 3:**
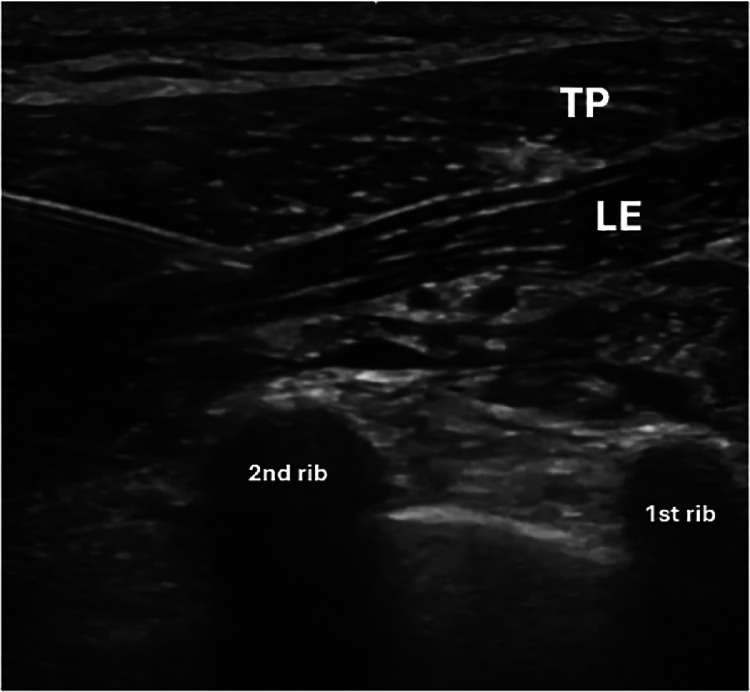
Needle positioned for LeSP block 1, demonstrating the approach to the interfascial space without injection. TP, trapezius muscle. LE, levator scapulae muscle.

**Fig. 4:**
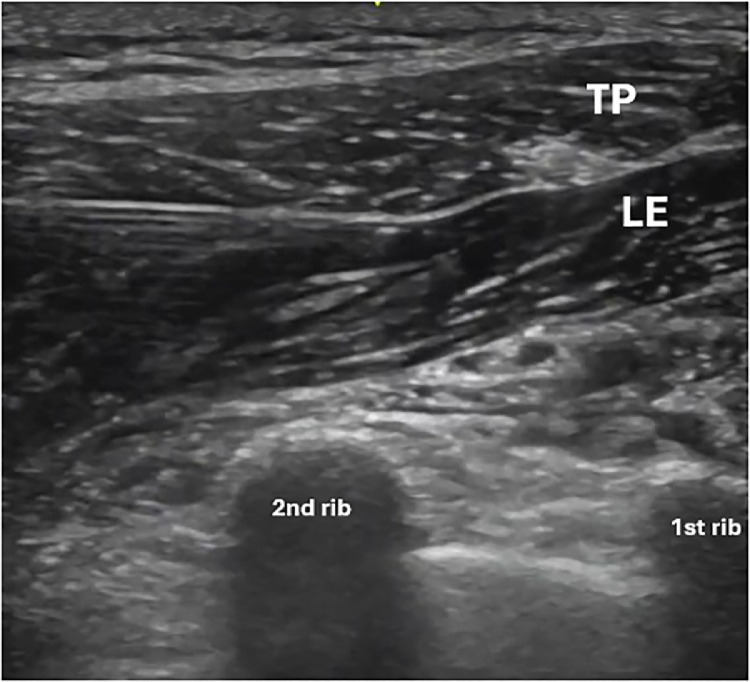
Administration of anesthetic into the interfascial space between the levator scapulae and surrounding structures, illustrating the spread of the solution in LeSP block 1. TP, trapezius muscle, LE, levator scapulae muscle.

This technique was named Levator Scapulae Plane (LeSPlane) Block. LeSP 1 is considered to be the anesthetic block of the superficial fascia, between the trapezius and levator scapula muscles. For better dispersion of the content, the interfascial space between the anterior edge of the trapezius to the proximal fourth was used to perform the block to disperse more content over the trapezius muscle without, however, allowing the content to leak into the anterior cervical branches, which could affect intrinsic neck muscles (Fig. 2, Fig. 3, Fig. 4).

**Fig. 5:**
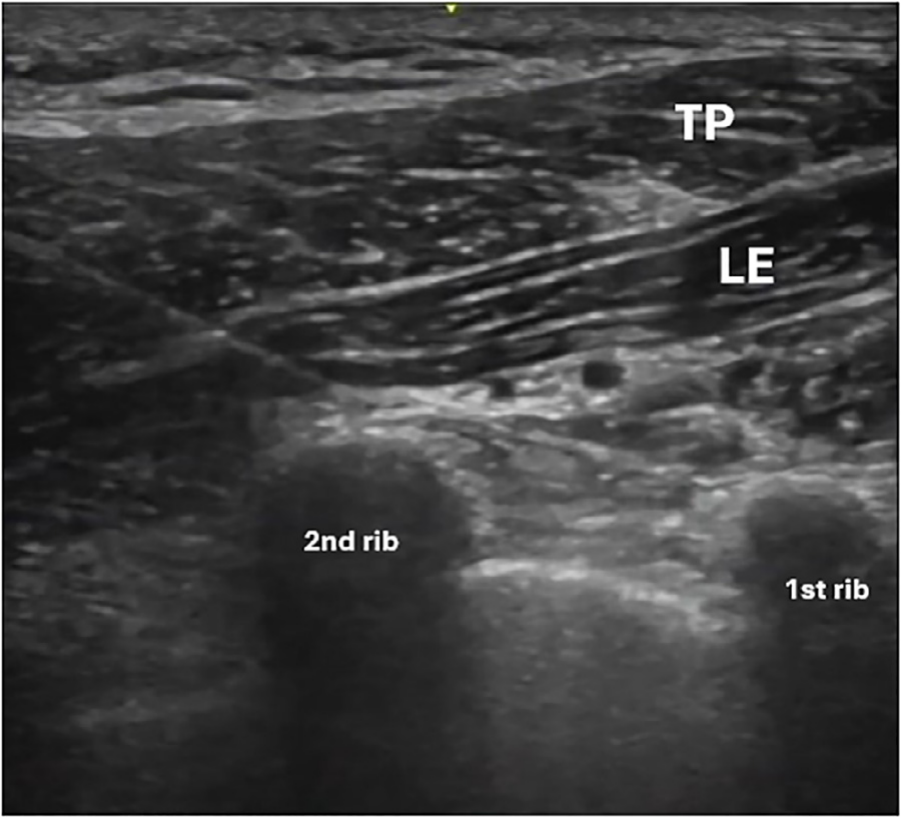
Needle placement for the LeSP block 2, showing the preinjection setup. TP, trapezius muscle, LE, levator scapulae muscle.

**Fig. 6:**
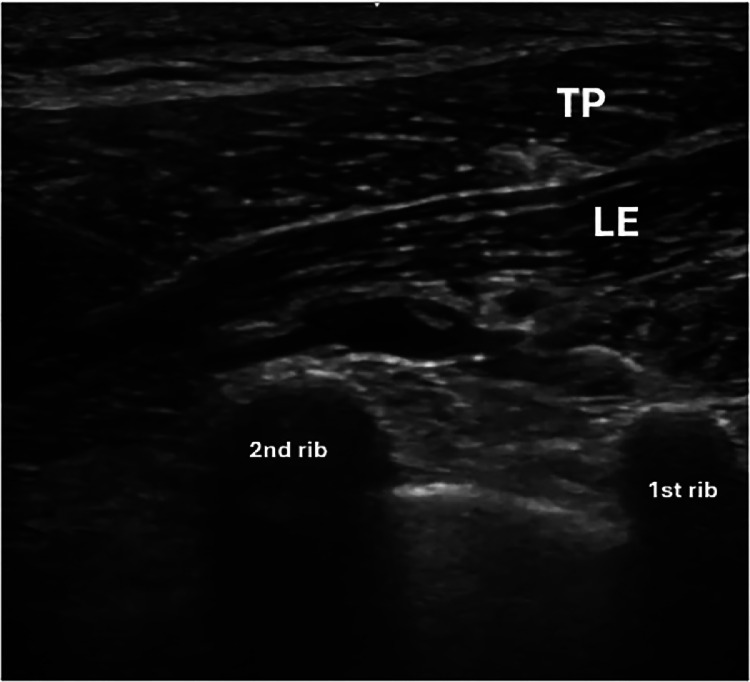
Injection of anesthetic into the interfascial space surrounding the levator scapulae, depicting the dispersion of the anesthetic solution in LeSP block 2. TP, trapezius muscle, LE, levator scapulae muscle.

LeSP 2 was named as the procedure that targets the space above the edge of the second rib, in which way the content would block the deep fascia and, by mass dispersion, block the dorsal nerve of the scapula with the safety of the bone of the second rib, thus avoiding the unnecessary risk of pneumothorax (Figs. 2, 5 and 6).

For the injection, a Pajunk® cannula type PKTL for sensory and motor stimulation and an Aprokorea® generator for sensory and motor stimulation were used. Sensitive stimuli were performed at each point with a frequency of 50Hz to 1volt reproducing irradiation in the pain path and motor stimulus at 2 hz with discrete movement of the levator scapulae and rhomboid muscles respectively. After confirmation at each site described above, the solution was injected observing the separation of the fascia.

## Discussion

Because surrounding conditions such as pain from the cervical spine or pain from the rotator cuff may predominate, the prevalence of pain in the shoulder girdle region due to myofascial causes may be underestimated. When a condition is identified, conservative treatments such as physiotherapy, diathermy, analgesic medications, muscle relaxants, and anti-inflammatory drugs are used [20,21].

Minimally invasive approaches include regional or distant acupuncture, dry needling, and inactivation of trigger points at the pain site, as well as cervical blocks when facet arthrosis or cervical spine foramen stenosis is suspected as the cause of myofascial pain [10,22,23]. Fascia blocks are performed in several places for regional anesthesia, including cervical and occipital pain, such as superficial cervical plexus block and occipital nerve block [24,25].

Although the trapezius muscle is innervated by the accessory spinal nerve, a branch of the XI pair, and branches of C2, the course differs from that of the superficial cervical plexus. A superficial cervical plexus block is performed in the anterolateral region of the neck by placing the needle tip below the lateral border of the sternocleidomastoid muscle at the height of the sixth cervical vertebra, C6. The cervical plexus arises from the anterior rami of the C1-C4 cervical spinal nerves. Thus, a cervical plexus block is properly defined as a blockage of the anterior branches of C2-C4, but it is not possible to consider that this block includes the sensory branches that innervate the trapezius muscle [26,27].

Pain management after superficial neck and shoulder procedures, thyroid and parathyroid surgery, and carotid endarterectomy are all common indications for a superficial approach to cervical plexus block [22].

The greater occipital nerve arises from the medial branch of the dorsal branch of the second cervical spinal nerve (C2). Greater occipital block is used to provide analgesia in patients undergoing posterior craniotomy and is best accomplished using selective nerve block techniques. In contrast, injections deep into the prevertebral fascia can cause phrenic or brachial plexus blockage or injury. From the vertex to the external occipital protuberance, the greater occipital nerve innervates the skin and fasciae of the posterior scalp [28,29].

### Ultrasound-guided technique

Due to location of the pain and the anatomical arrangement of the nerves, the mentioned block was chosen for both analgesia and safety. The first blocking point was in the superficial fascia and the technique was performed in the ultrasound plane, in the posteroanterior direction. In this manner, the needle tip is kept away from the pleura. At the second point of blockage, in the deep fascia, the levator scapulae is positioned on the long axis, leaving the first and second ribs on the ultrasound image. The dorsal artery of the scapula, which accompanies the dorsal nerve of the scapula, can be located in this region. The needle is introduced in plane, also in the posteroanterior direction, with the ribs guaranteeing additional safety from the risk of pneumothorax.

It is possible to block the 2 fascia using the same entry point with a 10 cm long needle, without having to remove the needle from the skin. The ideal image for blocking initially consists of presenting the trapezius muscle on the short axis, the levator scapulae muscle on the long axis, and the first and second ribs on the short axis on the screen. In this manner, the needle is introduced in the posteroanterior plane with the ultrasound, towards the anterior third of the fascia that separates the trapezius muscle from the levator scapulae muscle. Then, the needle is withdrawn until next to the skin without removing it and reintroduced in the same plane towards the second rib. At this point, it is worth turning on the ultrasound “colors” mode to avoid needle contact with the dorsal artery of the scapula.

Since LeSP Block is an innovative procedure being described for the first time, the sample size was small. Although there has been no variation in the ultrasound-guided technique so far, each case's postprocedure management, environmental conditions, and social support have been different, making it difficult to form a homogeneous group.

This study was based on the review of medical records, which limits the amount of data that can be collected (for example, only VAS as a quantitative parameter could be analyzed). In addition, the long term effectiveness of the procedure could be better observed if a longer follow-up time was considered. Also, fascial plane blocks present variability and some unpredictability regarding the pattern of sensory blockade, differing from traditional regional anesthesia blocks, which are nerve-specific. This may result in inconsistent outcomes depending on the anatomical variations and the extent of the spread of the anesthetic solution.

It is still difficult to define whether the accessory spinal nerve was assessed, so more studies involving cadaver dissections and anatomical analysis should be performed. This study is the first report of cases with measurement of results describing a new technique (LeSP Block 1 and 2). As this is an innovative technique, there have been no double-blind randomized controlled studies to date to evaluate the effectiveness of the treatment. Consequently, it was very difficult for the authors to safely determine anatomical parameters so as not to perform any iatrogenesis during the ultrasound guided procedures. The anatomical landmarks of the study were initially evidenced based on ultrasound examinations and procedures on cadavers in the anatomy laboratory of the medical school of the same hospital where the procedures were carried out.

The first descriptions of accessory spinal nerve block for trapezius myofascial pain appeared in 2011, but in a case report, the technique was performed with the assistance of a continuous local anesthetic drip catheter, making the technique unlikely to be performed in an outpatient setting. released on the same day [30]. The target of the described block was the spinal accessory nerve rather than the territory innervated by it. As a result, motor blockade of the intrinsic neck muscles is to be expected.

Trapezius fascia blockade was previously tested by Cho [31], however, comparing the superficial fascia blockade with regional pulsed radiofrequency neuromodulation. The results were positive with longer duration of the group submitted to pulsed radiofrequency. Following the trend of blocking the trapezius plane, other authors followed suit, finding similar results without associating it with the deep fascia. The block was effective for upper trapezius pain, as well as cervicogenic headache [12,13].

The approach of hydrodissection of the plane of the dorsal nerve of the scapula was also performed, but for a patient with shoulder girdle overload who developed paresthesia of the medial border of the scapula and was successful with the block [32]. However, because the technique used to locate the nerve between the ribs lacked precise bone reference or fascia, it was difficult to spread the method as a simple and safe option.

Considering the results obtained with the LeSP block, we can conclude that for pain limited to the upper and descending trapezius, performing only the block in the superficial plane, LeSP 1 (Fig. 4), is suggested and for pain that includes the rhomboid muscles and medial border of the scapula, the block of the deep fascia, LeSP 2 is recommended (Fig. 6). For pain in the entire region from the trapezius to the inferior angle of the scapula, it is then suggested to block the 2 fascia, superficial and deep of the levator scapula muscle, LeSP 1 and 2. Further studies are necessary, including an anatomical study to evaluate the spread of the solution following infiltration. Additional clinical trials are also required to better understand the efficacy, safety, and long-term outcomes of this technique in different patient populations.

Due to the procedure's safety and ease of use, as well as the results obtained, we recommend including the LeSP block in the therapeutic arsenal to control pain in the shoulder girdle, medial border of the scapula, and rhomboid muscles. More anatomical studies aimed at improving the anatomical description of the accessory spinal nerve should be carried out to improve the technique's accuracy.

## Data availability statement

The datasets generated during and/or analyzed during the current study are not publicly available due to rules of the hospital where the procedures were performed, which require confidentiality of patient data, as well as information security but are available from the corresponding author on reasonable request.

## Supplementary material

In the supplementary materials there are the terms of informed consent for patients number 1 and 2 described in the case report.

The CARE Checklist was provided to adapt the manuscript according to the established guidelines.

## Author contributions

**Roberto Del Valhe Abi Rached**, M.D, Ph.D.—Responsible for research planning and data collection, as well as data analysis and writing of the article. **Leandro Ryuchi Iuamoto**, M.D—Responsible for planning and data collection, as well as data analysis and review of article writing. **Angela Hyun Ji Kim**, M.D—Responsible for data collection, data analysis and also review of the discussion text. **Guilherme Yuiti Sikusawa**, M.D—Supervision and review of results, review of the discussion text and conclusion of the scientific article. **Fernanda Mayume Souza**, M.D—General supervision of the scientific article from its conception, writing, data collection and discussion among authors at each stage of the project. **Wu Tu Hsing**, M.D, Ph.D.—General supervision of the scientific article from its conception, writing, data collection, discussion among authors at each stage of the project and review of article writing.

## Patient consent

Participants signed an informed consent form approved by the ethics committee of the School of Medicine of the University of São Paulo, according to the Declaration of Helsinki Ethical Principles for Medical Research Involving Human Subjects.
